# Nutrients Intake Is Associated with DNA Methylation of Candidate Inflammatory Genes in a Population of Obese Subjects

**DOI:** 10.3390/nu6104625

**Published:** 2014-10-22

**Authors:** Valentina Bollati, Chiara Favero, Benedetta Albetti, Letizia Tarantini, Alice Moroni, Hyang-Min Byun, Valeria Motta, Diana Misaela Conti, Amedea Silvia Tirelli, Luisella Vigna, Pier Alberto Bertazzi, Angela Cecilia Pesatori

**Affiliations:** 1 Center of Molecular and Genetic Epidemiology, Department of Clinical Sciences and Community Health, Università degli Studi di Milano, Milan 20122, Italy; E-Mails: chiara.favero@unimi.it (C.F.); benedetta.albetti@unimi.it (B.A.); letizia.tarantini@unimi.it (L.T.); alice.moroni1@gmail.com (A.M.); valeria.motta@unimi.it (V.M.); pieralberto.bertazzi@unimi.it (P.A.B.); angela.pesatori@unimi.it (A.C.P.); 2 Epidemiology Unit, Fondazione IRCCS Ca’ Granda Ospedale Maggiore Policlinico, Milan 20122, Italy; 3 Laboratory of Environmental Epigenetics, Exposure Epidemiology and Risk Program, Harvard School of Public Health, Boston, MA 02115, USA; E-Mail: hmbyun@hsph.harvard.edu; 4 Worker’s Health Protection and Promotion Unit, Fondazione IRCCS Ca’ Granda Ospedale Maggiore Policlinico, Milan 20122, Italy; E-Mails: diana.misaela@gmail.com (D.M.C.); luisella.vigna@policlinico.mi.it (L.V.); 5 Laboratory of Clinical Chemistry & Microbiology, Fondazione IRCCS Ca’ Granda Ospedale Maggiore Policlinico, Milan 20122, Italy; E-Mail: amedea.tirelli@policlinico.mi.it

**Keywords:** DNA methylation, CD14, Et-1, iNOS, HERV-w, TNFα, nutrients

## Abstract

The aim of the present study was to evaluate the potential association between dietary nutrients and alterations in DNA methylation in a set of five candidate genes, including CD14, Et-1, iNOS, HERV-w and TNFα, in a population of overweight/obese subjects. We evaluated possible associations between gene methylation and clinical blood parameters, including total cholesterol (TC), low- and high-density lipoprotein cholesterol (LDL-C and HDL-C), triglyceride and homocysteine levels. We employed validated methods to assess anthropometric, clinical and dietary data, as well as pyrosequencing to evaluate DNA methylation of the five candidate genes in 165 overweight/obese subjects. There was no association between body mass index and DNA methylation of the five candidate genes in this group of subjects. Positive associations were observed between TNFα methylation and blood levels of LDL-C (β = 0.447, *p* = 0.002), TC/HDL-C (β = 0.467, *p* = 0.001) and LDL-C/HDL-C (β = 0.445, *p* = 0.002), as well as between HERV-w methylation and dietary intakes of β-carotene (β = 0.088, *p* = 0.051) and carotenoids (β = 0.083, *p* = 0.029). TNFα methylation showed negative associations with dietary intakes of cholesterol (β = −0.278, *p* = 0.048), folic acid (β = −0.339, *p* = 0.012), β-carotene (β = −0.332, *p* = 0.045), carotenoids (β = −0.331, *p* = 0.015) and retinol (β = −0.360, *p* = 0.008). These results suggest a complex relationship among nutrient intake, oxidative stress and DNA methylation.

## 1. Introduction

Obesity is characterized by chronic low-grade inflammation and immune system activation, which may contribute to the pathogenesis of obesity-related disorders [1,2,3,4]. Diet is a key factor in lifestyle habits. Dietary nutrition has been widely investigated in an attempt to understand its impact on health and disease status [5,6,7,8]. Poor diet may be associated with an increased risk of developing chronic diseases via the production of reactive oxygen species (ROS), which may lead to oxidative DNA damage [9,10,11].

In recent years, various efforts have been made towards evaluating the ability of antioxidant nutrients to limit the inflammatory process. Carotenoids have been shown to act as free radical scavengers that are capable of limiting the oxidation of low-density lipoprotein cholesterol (LDL-C) [12,13]. Examples of low molecular weight dietary antioxidants include vitamin A (retinol), vitamin C and vitamin E [14]. When endogenous antioxidant levels are no longer protective, ROS levels increase, leading to alterations in the normal redox state of cells and increased oxidative stress [14].

However, as there is no “universal” antioxidant and each antioxidant reacts differently with various ROS to generate final products with variable reactivity, the connections between antioxidant nutrients and inflammation are complex [15]. Pro-oxidant treatment may also be useful, as low levels of pro-oxidants may exert a mild stress signal that could, in turn, lead to increased levels of endogenous antioxidants [15].

In this context, the term “antioxidant paradox” [16] is often used to refer to the observation that although certain ROS have been linked to several human diseases, large doses of dietary antioxidant supplements may result in little to no preventive or therapeutic effect.

There is a close link between oxidative DNA damage and DNA methylation, which is a dynamic epigenetic regulatory mechanism that controls gene expression by inhibiting the binding of transcription machinery to DNA and changing the structure of the associated chromatin [17].

Oxidative lesions interfere with the ability of DNA to serve as an optimal substrate for the DNA methyltransferase protein, leading to global hypomethylation [18]. Epigenetic processes are dynamic, reversible and susceptible to exogenous factors. Therefore, they offer a unique opportunity for dietary-based interventions that target epigenetic pathways [19].

The aim of the present study was to evaluate alterations in DNA methylation induced by dietary nutrients in a population of overweight/obese subjects. In particular, we measured DNA methylation in a set of five candidate genes, including CD14, endothelin-1 (Et-1), inducible nitric oxide synthase (iNOS), human endogenous retrovirus type w (HERV-w) and tumor necrosis factor alpha (TNFα), based on the important roles that these genes play in the inflammation and oxidative stress processes. Furthermore, we evaluated possible associations between the methylation of these candidate genes and blood clinical parameters, including total cholesterol (TC), LDL-C, high-density lipoprotein cholesterol (HDL-C), triglyceride (TG) and homocysteine (HC) levels.

The results of this study uncover a set of potentially informative molecular markers and provide a biological basis for the critical evaluation of clinical studies whose results suggest that a balanced diet may be capable of reducing disease risk and incidence in the general population.

## 2. Experimental Section

### 2.1. Study Subjects

Study subjects included 165 overweight/obese subjects older than 18 years who were recruited at the Center for Obesity and Work (Department of Preventive Medicine, Istituti di Ricovero e Cura a Carattere Scientifico (IRCCS), Fondazione Ca’Granda, Ospedale Maggiore Policlinico during the period between September, 2010, and April, 2011.

Weight and height were measured by a nurse following standardized procedures. Body mass index (BMI) was calculated as the ratio between the subject’s weight (kg) and height squared (m^2^). According to the current definition [20], subjects who had a BMI of between 25 and 29.9 were classified as overweight, and subjects who had a BMI of 30 or higher were classified as obese. Waist circumference (cm) was also assessed as a marker of central fat accumulation.

Exclusion criteria included: pregnancy, previously diagnosed cancers, heart disease or stroke during the year before enrollment and other chronic diseases, such as autoimmune diseases, multiple sclerosis, Alzheimer disease, Parkinson disease, depression, bipolar disorder, schizophrenia and epilepsy. Subjects who reported a diet protocol in the last year or a substantial change in physical activity during the month before the study began were also excluded (seven subjects). The participation rate was 90%.

Each participant signed an informed consent form, which had been approved by the Ethics Committee of the Fondazione Ca’Granda, Ospedale Maggiore Policlinico (Approval Number 1425), in accordance with the principles of the Declaration of Helsinki. Subjects who agreed to participate were asked to complete lifestyle and dietary questionnaires and to donate a blood sample. Routine data were collected from each subject, including fasting levels of TC, HDL-C, LDL-C, TG (mg/dL), HC (µmol/L), glucose and postprandial blood glucose and measured from serum samples through standard protocols (Modular, Roche, Basel, Switzerland).

### 2.2. Lifestyle Factors and Dietary Assessment

Information about lifestyle factors, including smoking, alcohol consumption and physical activity, were collected with a self-administered questionnaire.

A standardized food frequency questionnaire (FFQ) validated for the Italian population [21,22] was used to assess the typical dietary intake (according to an average standard portion) of each study participant. Table S1 describes food categories assessed by the FFQ. Frequency of food consumption was recorded on weekly and monthly bases.

**Table 1:** Pyrosequencing assay information.

Gene	Chromosome	CpG Position *	Primers: Forward (F), Reverse (R), and Sequencing (S)
**CD14**	5	140632047	**F**: GGGTTTATAGAGGAGGGAATTGA
		140632043	**R**: Biotin-AAACCCCATCCAACCCCTAT
		140632041	**S**: TGTAGGGTTTTGGGG
		140632031	
**iNOS**	17	27799241	**F**: TTAGGGTTAGGTAAAGGTATTTTTGTTT
		27799233	**R**: Biotin-CAATTCTATAAAACCACCTAATAATCTTAA
			**S**: TAAAGGTATTTTTGTTTTAA
**Et-1**	6	12290364	
		12290366	**F**: TTGTTTGGGGTTGGAATAAAGT
		12290380	**R**: Biotin-ATCCTTCAACCCAAATACCCTTTT
		12290387	**S**: GGTAGAGAGTTGTTTAAGTT
		12290391	
**HERV-w**	7	92468887	**F**: AAGTTATAGTTGAAGGAAGA
		92468872	**R**: Biotin-CAATCCCCCATCTCAACAA
		92468856	**S**: AGTTTAAGATTAAGATTTAT
**TNFα**	6	2874672	**F**: Biotin-TGAGGGGTATTTTTGATGTTTGT
		2874678	**R**: TTAATAATTATTTTTATATATTTT
		2874680	**S**: ATAAATTTTATATTTTTTAT
		2874695	

* According to the University of California, Santa Cruz, UCSC Genome Browser, December, 2013, release (GRch38/hg38) [23].

Nutrient intake and diet composition were calculated with the “Osservatorio Nutrizionale Grana Padano” (ON-GP) [24]. This online tool was launched in 2005, with the aim of developing an innovative tool for nutritional education and epidemiological monitoring of the Italian general population.

### 2.3. Sample Collection, DNA Extraction and Bisulfite Treatment

Seven milliliters of whole blood were collected into EDTA tubes from each participant by venous phlebotomy. Blood was centrifuged at 2,500 rpm for 15 min. The buffy coat fraction was transferred to a cryovial and immediately frozen at −80 °C until use. DNA was extracted by the Wizard Genomic DNA purification kit (Promega, Madison, WI, USA), according to the manufacturer’s instructions.

Genomic DNA (500 ng total) was treated with the EZ DNA Methylation-Gold™ Kit (Zymo Research, Orange, CA, USA), in accordance with the manufacturer’s protocol. Bisulfite-treated DNA was eluted in 30 μL of M-Elution Buffer.

### 2.4. DNA Methylation

Analysis of DNA methylation was performed by previously published methods [25,26], with minor modifications. Briefly, a 50-μL PCR reaction was carried out with 25 μL of GoTaq Green Master mix (Promega), 1 pmol of forward primer, 1 pmol of biotinylated reverse primer and 500 ng of bisulfite-treated genomic DNA. PCR cycling conditions and primer sequences are provided in Table 1. Biotin-labeled primers were used to purify the final PCR product with Sepharose beads. The PCR product was bound to a Streptavidin Sepharose HP (Amersham Biosciences, Uppsala, Sweden). Sepharose beads containing the immobilized PCR product were purified, washed, denatured with 0.2 M NaOH and washed again with the Pyrosequencing Vacuum Prep Tool (Pyrosequencing, Inc., Westborough, MA, USA), according to the manufacturer’s instructions. Pyrosequencing primer (0.3 μM) was annealed to the purified single-stranded PCR product, and pyrosequencing was performed with the PyroMark MD System (Pyrosequencing, Inc. Westborough, MA, USA). The degree of methylation was expressed as the percentage of cytosines that were methylated, determined as the number of methylated cytosines divided by the sum of methylated and unmethylated cytosines, multiplied by 100% (% 5-methyl-Cytosine). Every sample was tested three times for each marker to confirm reproducibility and to increase the precision of the findings.

### 2.5. Statistical Analysis

The data were summarized with standard descriptive statistics. Categorical variables are presented as absolute numbers and frequencies. Continuous variables are expressed as the mean ± standard deviation (SD) or as the median (first quartile–third quartile). Normality was verified by graphical inspection (e.g., normality plot). A *p*-value of 0.05 was considered statistically significant. All analyses were performed in SAS 9.3 (SAS Institute Inc., Cary, NC, USA).

To estimate the effect of BMI, nutrient intake and blood clinical parameters on DNA methylation, linear mixed-effect models were fitted using PROC MIXED in SAS v 9.3.

DNA methylation measurements for each subject were run in duplicate (Runs 1 and 2). The pyrosequencing-based DNA methylation analysis tests a variable number of CpG positions, according to CpG density in the promoter assay: eight values for CD14 (methylation at four CpG dinucleotide positions replicated in two measurements), 10 values for Et-1, six values for HERV-w, four values for iNOS and eight values for TNFα.

A linear mixed-effect model was used to account for each CpG dinucleotide position measured in the two runs and the potential confounding effect of the plate. An unstructured covariance structure was used to model within-subject errors. The Kenward–Roger approximation was used to estimate the degrees of freedom in the denominator. DNA methylation was treated as a continuous variable, and the CpG site was considered a random effect.

Multivariable models were adjusted for age, sex, BMI (only for models with nutrient and blood clinical parameters), cigarette smoking (never, former and current) and neutrophil percentage. The roles of other possible covariates were evaluated through univariate analyses, but none of the covariates resulted in an association with DNA methylation. Scaled regression coefficients were used to represent the increase in DNA methylation estimated for a 1-SD increment in the independent variable.

## 3. Results

A description of the study population is reported in Table 2. Study participants were predominantly female (84% of population). Fifty-four participants were overweight (eight males and 46 females), and 111 participants were obese (25 males and 86 females). The mean age of the study population was 50.3 years (11.5 years). The mean BMI was 33.6 kg/cm^2^ (6.0 kg/cm^2^), and the mean waist circumference was 100.4 cm (13.5 cm).

**Table 2:** Baseline subjects characteristics.

Characteristic	*n*	Mean ± SD	Median [Q1–Q3]
Age, years	165	50.3 ± 11.5	51.9 [43.3–58.5]
Sex, *n* (%)			
Male	33 (20.0%)		
Female	132 (80.0%)		
BMI, kg/cm^2^	165	33.6 ± 6.0	32.9 [29.2–37.3]
Male	33	34.0 ± 4.9	33 [30–38.8]
Female	132	33.5 ± 6.3	32.8 [29.1–37.2]
25–30 (overweight)	54 (32.7%)		
≥30 (obese)	111 (67.3%)		
Waist circumference, cm	164	100.4 ± 13.5	99 [90–109]
Male	33	110.1 ± 12	109 [104–115]
Female	132	98.1 ± 12.8	96 [89–104]
Cholesterol, mg/dL	163	220.3 ± 41.1	215 [196–243]
Cholesterol LDL, mg/dL	163	137.1 ± 34.3	134 [116–158]
Cholesterol HDL, mg/dL	163	59.5 ± 14.3	58 [50–69]
Total cholesterol/HDL ratio			
Male	33	4.3 ± 1.1	4.2 [3.5–4.9]
Female	130	3.8 ± 1.1	3.6 [3–4.3]
LDL cholesterol/HDL ratio			
Male	33	2.8 ± 0.9	2.8 [2.2–3.3]
Female	130	2.4 ± 0.8	2.2 [1.8–2.8]
Triglycerides, mg/dL	163	113.8 ± 64.5	97 [75–139]
Fasting glucose, mg/dL	161	96.9 ± 25	91 [86–101]
Post prandial blood glucose, mg/dL	163	106 ± 29.7	97 [90–112]
Homocysteine, μmol/L	158	10.8 ± 4.4	9.9 [8.2–12.3]
Smoking, * n* (%)			
Never smoked	86 (52.1%)		
Ex-smoker	50 (30.3%)		
Actual smoker	24 (14.6%)		
Missing	5 (3.0%)		
Physical activity frequency, *n* (%)			
Never	84 (50.9%)		
<2 times a week	25 (15.2%)		
2–4 times a week	19 (11.5%)		
>4 times a week	21 (12.7%)		
Missing	16 (9.7%)		

Among the study participants, 52% had never smoked, 30.3% were former smokers and 14.6% were current smokers. Information about smoking status was missing for five participants. Approximately 50% (84 participants) of participants reported no activity; 15.2% (25 participants) reported an activity frequency of less than two times/week; 11.5% (19 participants) reported an activity frequency of two to four times/week; and 12.7% (21 participants) reported an activity frequency of more than four times/week. The mean TC level was 220.3 mg/dL (41.1 mg/dL); the mean total LDL-C level was 137.1 mg/dL (34.3 mg/dL); the mean total HDL-C level was 59.5 mg/dL (14.3 mg/dL); the median TG level was 97 mg/dL (75–139 mg/dL); and the median HC level was 9.9 μmol/L (8.2–12.3 μmol/L).

Table 3 describes the nutrient and diet composition for the study population. Recommended average intake of selected food nutrients are reported in Table S2.

**Table 3:** Nutrients composition. Continuous variables are expressed as the mean ± SD or as the median (25th percentile–75th percentile).

Nutrient	*n*	Mean ± SD	Median [Q1–Q3]
Fiber, g/day	165	20.7 ± 8.22	19.34 [15.08–25.95]
Protein, g/day	165	85.42 ± 27.84	81.25 [67.03–100.27]
Carbohydrate, g/day	165	213.62 ± 78.98	209.51 [154.39–254.18]
Lipids, g/day	165	63.52 ± 27.5	59.92 [43.49–77.55]
Monounsaturated fatty acid (MUFA), g/day	131	26.16 ± 11.41	24.44 [18.78–31.8]
Polyunsaturated fatty acid (PUFA), g/day	131	12.39 ± 4.52	11.96 [9.18–15.05]
PUFA n-3, g/day	165	1.13 ± 0.41	1.04 [0.89–1.41]
Saturated fatty acid, g/day	165	25.59 ± 11.36	23.64 [17.02–32]
Cholesterol , mg/day	165	266 ± 101	260.21 [201.69–324.85]
Ascorbic acid, mg/day	165	149.01 ± 76.03	135.7 [100.23–196.02]
Folic acid, μg/day	165	301.45 ± 133.09	279.07 [205.7–363.11]
α-carotene, μg/day	131	1,006.24 ± 1,145.61	620.31 [157.87–1,310.38]
β-carotene, μg/day	131	6,845.55 ± 4,867.2	5,626.83 [3,300.42–8,952.27]
Carotenoids, μg/day	165	20,893.62 ± 13,519.3	17,527.85 [11,884.24–26,177.22]
Polyphenols and flavonoids, mg/day	131	90.09 ± 70.68	73.57 [39.42–127.4]
Retinol, μg/day	165	1,623.05 ± 1,011.97	1,333.7 [961.1–2,057.6]
Tocopherols, mg/day	165	8.78 ± 3.2	8.5 [6.85–10.74]
Vitamin B12, μg/day	165	5.95 ± 2.17	5.47 [4.52–7.18]
Vitamin D, μg/day	165	3.86 ± 2.46	3.37 [2.41–4.92]

We did not observe any significant associations between BMI and DNA methylation of CD14, iNOS, Et-1, HERV-w or TNFα when we used a multivariable mixed-effect model adjusted for age, sex, smoking status and neutrophil percentage (Table 4). Similar results were obtained when being overweight/obesity was included as a dichotomous variable in the model (data not shown). We did not identify any association between waist circumference and DNA methylation of any of the five candidate genes using this same approach (data not shown).

**Table 4:** Association of BMI with blood gene methylation.

Methylation	β *	SE	*p*-value
CD14	0.033	0.137	0.807
Et-1	−0.056	0.056	0.317
HERV-w	−0.043	0.038	0.260
iNOS	−0.447	0.399	0.264
TNFα	−0.154	0.135	0.258

Multivariable mixed-effect models adjusted for age, sex, smoking and % of neutrophils. * β regression coefficients were scaled to represent the increase in the dependent variable estimated for an increment equal to 1 SD in the independent variable.

**Table 5:** Association of the blood clinical parameters with blood gene methylation.

Blood Clinical Parameters	CD14			Et-1			HERV-W			iNOS			TNFα		
	β *	SE	*p*-value	β *	SE	*p*-value	β *	SE	*p*-value	β *	SE	*p*-value	β *	SE	*p*-value
Triglycerides, mg/dL	−0.004	0.147	0.977	−0.002	0.060	0.972	0.026	0.041	0.533	−0.571	0.425	0.182	0.212	0.145	0.144
Homocysteine, µmol/L	0.036	0.140	0.797	0.056	0.056	0.324	−0.017	0.039	0.662	0.590	0.407	0.150	0.142	0.138	0.306
Cholesterol LDL, mg/dL	0.257	0.146	0.080	0.054	0.060	0.369	0.019	0.042	0.648	0.450	0.430	0.297	0.447	0.143	0.002
Cholesterol HDL, mg/dL	0.154	0.142	0.280	−0.016	0.058	0.782	−0.042	0.040	0.301	−0.014	0.418	0.974	−0.205	0.141	0.147
Total cholesterol/HDL ratio	0.092	0.144	0.525	0.050	0.058	0.392	0.042	0.041	0.307	0.327	0.420	0.438	0.467	0.139	0.001
LDL cholesterol/HDL ratio	0.087	0.143	0.541	0.067	0.057	0.247	0.044	0.040	0.273	0.78	0.417	0.366	0.445	0.138	0.002

Multivariable mixed-effect models adjusted for age, sex, BMI, smoking and % of neutrophils. * β regression coefficients were scaled to represent the increase in the dependent variable estimated for an increment equal to 1 SD in the independent variable.

**Table 6:** Association of nutrients with blood gene methylation.

Nutrients	CD14			Et-1			HERV-w			iNOS			TNFα		
	β *	SE	*p*-value	β *	SE	*p*-value	β *	SE	*p*-value	β *	SE	*p*-value	β *	SE	*p*-value
Fiber, g/day	−0.034	0.140	0.808	−0.028	0.058	0.628	0.054	0.039	0.164	0.160	0.408	0.695	−0.182	0.139	0.190
Protein, g/day	−0.012	0.142	0.935	0.001	0.059	0.984	0.029	0.039	0.459	−0.328	0.413	0.429	−0.234	0.140	0.097
Carbohydrate, g/day	−0.058	0.145	0.691	−0.016	0.060	0.786	−0.008	0.040	0.848	−0.134	0.424	0.752	−0.080	0.145	0.583
Lipids, g/day	−0.186	0.141	0.190	0.001	0.059	0.982	0.007	0.040	0.865	0.095	0.416	0.819	−0.157	0.141	0.269
Monounsaturated fatty acid (MUFA), g/day	−0.228	0.170	0.182	−0.033	0.063	0.599	0.009	0.046	0.837	−0.206	0.438	0.638	−0.208	0.166	0.213
Polyunsaturated fatty acid (PUFA), g/day	−0.233	0.170	0.174	−0.044	0.065	0.495	0.009	0.046	0.850	−0.172	0.440	0.697	−0.199	0.168	0.239
PUFA *n*-3, g/day	−0.186	0.143	0.196	0.001	0.059	0.987	0.009	0.040	0.825	0.147	0.420	0.726	−0.117	0.143	0.414
Saturated fatty acid, g/day	0.098	0.141	0.489	0.080	0.059	0.176	0.011	0.039	0.772	−0.693	0.409	0.092	−0.183	0.140	0.192
Cholesterol , mg/day	−0.054	0.142	0.703	−0.022	0.059	0.713	0.021	0.039	0.590	−0.221	0.414	0.595	−0.278	0.139	0.048
Ascorbic acid, mg/day	0.230	0.138	0.098	−0.022	0.058	0.700	0.072	0.039	0.064	0.155	0.408	0.704	−0.146	0.139	0.295
Folic acid, μg/day	0.159	0.135	0.242	0.038	0.056	0.501	0.067	0.038	0.079	−0.203	0.398	0.610	**−0.339**	**0.133**	**0.012**
Alpha carotene, μg/day	0.050	0.170	0.770	−0.013	0.062	0.840	0.040	0.045	0.380	0.401	0.435	0.359	−0.190	0.165	0.253
Beta carotene, μg/day	0.170	0.169	0.316	0.027	0.063	0.673	**0.088**	**0.044**	**0.051**	0.210	0.436	0.631	**−0.332**	**0.164**	**0.045**
Carotenoids, μg/day	0.124	0.137	0.368	0.044	0.056	0.436	**0.083**	**0.037**	**0.029**	0.102	0.402	0.801	**−0.331**	**0.135**	**0.015**
Polyphenols and flavonoids, mg/day	0.156	0.173	0.370	−0.016	0.064	0.808	0.059	0.046	0.197	0.088	0.446	0.843	−0.152	0.169	0.371
Retinol, μg/day	0.103	0.137	0.453	0.026	0.056	0.645	0.071	0.038	0.063	0.026	0.403	0.949	**−0.360**	**0.134**	**0.008**
Tocopherols, mg/day	−0.081	0.138	0.556	−0.001	0.057	0.987	0.044	0.038	0.253	−0.002	0.403	0.996	−0.205	0.137	0.136
Vitamin B12, μg/day	0.044	0.143	0.758	−0.018	0.059	0.763	0.028	0.040	0.487	−0.021	0.417	0.960	−0.153	0.142	0.281
Vitamin D, μg/day	0.228	0.138	0.101	0.078	0.057	0.171	0.008	0.039	0.828	−0.844	0.401	0.037	−0.180	0.137	0.193

Multivariable mixed-effect models adjusted for age, sex, BMI, smoking and % of neutrophils. * β regression coefficients were scaled to represent the increase in the dependent variable estimated for an increment equal to 1 SD in the independent variable.

Table 5 shows the results of multivariable analyses adjusted for age, sex, BMI, smoking status and neutrophil percentage. For each clinical parameter, β regression coefficients are reported. TNFα methylation was positively associated with levels of LDL-C (*p* = 0.002), TC/HDL-C (*p* = 0.001) and LDL-C/HDL-C (*p* = 0.002). A similar model was run to evaluate the influence of selected dietary nutrients on DNA methylation (Table 6). We observed positive associations between HERV-w methylation and intakes of β-carotene (*p* = 0.051) and carotenoids (*p* = 0.029). TNFα methylation was negatively associated with intakes of dietary cholesterol (*p* = 0.048), folic acid (*p* = 0.012), β-carotene (*p* = 0.045), carotenoids (*p* = 0.015) and retinol (*p* = 0.008).

Diabetes may influence the inflammatory status of study subjects. In our study population, 14 individuals were diagnosed with diabetes, based on the recent American Diabetes Association Criteria [27]. The results did not change when we conducted sensitivity analyses that excluded these subjects (data not shown).

## 4. Discussion

We evaluated the effects of nutrient intake on the DNA methylation of five candidate inflammatory genes, CD14, Et-1, iNOS, HERV-w and TNFα, in 165 overweight/obese subjects who provided detailed information about their typical diet. We did not identify an association between BMI and the DNA methylation of any of the five candidate genes in our study population. TNFα methylation was positively associated with serum LDL-C, the TC/HDL-C ratio and the LDL-C/HDL-C ratio. Using data derived from the FFQ, we identified significant positive associations of HERV-w methylation with dietary intakes of β-carotene and carotenoids, as well as negative associations of TNFα methylation with dietary intakes of cholesterol, folic acid, β-carotene, carotenoids and retinol.

The observed methylation differences were small when expressed as a percentage of the total number of cytosines in the considered position. However, these differences involved a large number of DNA molecules and correspond to a large number of blood cells in which candidate gene expression might be altered.

When identifying candidate genes to be assessed for DNA methylation, we focused on genes with established roles in inflammation-related processes, genes whose expression levels are regulated by DNA methylation and genes for which a link to environmental exposure has been assessed. CD14 is a recognition receptor for environmental endotoxins, such as lipopolysaccharide. CD14 is expressed on the membranes of monocytes, macrophages and neutrophils or as a monocyte/liver-derived soluble serum protein [28]. CD14 expression is regulated by DNA methylation [29]. The expression and activity of iNOS (also known as NOS2) are increased in the presence of ROS [30]. Lower levels of DNA methylation in the iNOS promoter have been associated with increased expression [31]. Et-1 has proinflammatory activity in human circulating leukocytes [32], and its expression is upregulated after demethylation [33]. HERVs represent sequences descended from ancient viral infections that have been integrated into the human genome. HERV sequences can be transcriptionally active and are regulated by DNA methylation [34]. HERV-w elements encode a powerful immunopathogenic envelope protein that activates a proinflammatory autoimmune cascade via its interaction with Toll-like receptor 4 [35,36]. TNFα is a proinflammatory cytokine whose levels are commonly elevated in obese subjects. The TNFα promoter is susceptible to regulation by cytosine methylation [37]. TNFα has been demonstrated to be modified in response to the intake of several foods or nutrients, such as fruits and vegetables [38], olive oil and walnuts [39], fructose [40] and polyunsaturated fatty acids [41].

Obese/overweight subjects were recruited at the Center for Obesity and Work. Hence, the study population may be quite different from the general population, and these differences could inhibit the generalization of our findings. Notably, this population is characterized by a higher BMI and an average daily micronutrient intake that is higher than the average Italian daily intake. In addition, although the prevalence of overweight/obese males is higher than the prevalence of overweight/obese females in the general population, our study group included a higher proportion of overweight/obese women. One possible explanation is that women are, in general, more concerned about their appearance than men, and women may be more likely to seek out personalized diet protocols and diet centers that provide periodic medical checks.

Taking into account these limitations, our study is one of only a few that evaluates the association of BMI with the methylation of specific proinflammatory genes. We did not observe any significant association between DNA methylation of the candidate genes and BMI or waist circumference values in our overweight/obese population. One recent paper reported a negative association between TNFα methylation and BMI/total body fat in young women of normal weight [41]. The lack of coherent findings may be due, in part, to the diverse study populations: our study group included mostly middle-aged individuals of both sexes and only overweight/obese subjects. It is reasonable to speculate that normal-weight subjects may maintain a control mechanism for inflammation, mediated by BMI. This mechanism could be lost in obese subjects, who maintain a chronic low-level inflammatory state.

Our findings suggest an apparent contrasting role of dietary cholesterol intake *versus* serum cholesterol levels with regard to TNFα methylation. Cholesterol intake estimated through the diet questionnaire was negatively associated with TNFα methylation, whereas serum cholesterol levels were positively associated with TNFα methylation. Recent evidence suggests that dietary cholesterol may play a role in obesity-associated inflammation by directly exacerbating local inflammation in adipose tissue. This exacerbation might lead to cytokine-mediated inflammatory changes in the liver, systemic inflammation and atherosclerosis [42]. However, this hypothesis was not confirmed by the positive associations we identified between TNF-α DNA methylation and the serum LDL-C levels, the TC/HDL-C ratio and the LDL-C/HDL-C ratio. Even if the serum cholesterol measure is only due in small part to dietary cholesterol intake, we cannot fully explain this inconsistency.

β-carotene and carotenoids are considered beneficial dietary substances [12,13]. Their consumption in the study population was associated with a higher level of HERV-w DNA methylation, suggesting that reduced HERV-w expression may have a proinflammatory effect. β-carotene, carotenoids and retinol, which is another powerful antioxidant, were associated with reduced TNFα DNA methylation. Current data from the literature are conflicting: some studies have reported a positive relationship between TNFα DNA methylation and health effects, whereas others did not find any effect or even pro-oxidant effects (reviewed in [12]). Antioxidants and pro-oxidants encompass a wide range of related biological pathways. It could be difficult to interpret the direct effect of this fine balance on the methylation of a single gene. In addition, the difference that we observed is in agreement with recent evidence that pro-oxidant species are associated with tissue damage in chronic inflammatory diseases, but can also act to help resolve inflammation [15].

Nutrient quantification was based on a standardized self-administered FFQ that was developed to collect information about food items specific to the Italian population [21,22]. A limitation of this questionnaire is that it is only semiquantitative, because the questions ask how many times per week or per month specific foods are consumed. FFQs have been the most frequently used dietary assessment method in epidemiological studies, because they are easy to administer, have a relatively low cost and provide a rapid estimate of typical food intake. Nutritional values reported from FFQ data may be subject to errors, which have been analyzed extensively in nutritional epidemiology studies [43,44]. A specific limitation of FFQs is that derived micronutrient quantification is highly dependent on the difficulty associated with defining and describing a standard portion, especially when FFQs are self-administered.

## 5. Conclusions

The present exploratory study confirms the highly complex relationship between pro-oxidant and antioxidant compounds, which has emerged in recent studies. Further studies using larger sample sizes and a genome-wide approach are warranted to help increase our understanding of the complex relationship among food intake, oxidative stress and DNA methylation.

## Supplementary Files

Supplementary File 1
