# Supplementary material for: Nutrients Intake Is Associated with DNA Methylation of Candidate Inflammatory Genes in a Population of Obese Subjects
Source: Nutrients. 2014 Oct 22;6(10):4625–39. doi: 10.3390/nu6104625 (PMC4210937; doi:10.3390/nu6104625)
Supplement: Supplementary File 1 [file nutrients-06-04625-s001.docx]

**Supplementary Information**

**Table S1.** Information on usual diet collected by questionnaire.

# DIET

Which of the following foods consumed habitually and with what frequencies?

| FOODS | NUMBER OF TIMES A WEEK | NUMBER OF TIMES A MONTH |
| --- | --- | --- |
| STUFFED EGG PASTA | \|__\|__\| | \|__\|__\| |
| PASTA, RICE | \|__\|__\| | \|__\|__\| |
| SOUP (“Minestrone”) WITH PASTA | \|__\|__\| | \|__\|__\| |
| BREAD | \|__\|__\| | \|__\|__\| |
| POLENTA | \|__\|__\| | \|__\|__\| |
| PIZZA | \|__\|__\| | \|__\|__\| |
| WHOLE BREAD | \|__\|__\| | \|__\|__\| |
| CRACKERS, RUSKS, BREAD STICKS | \|__\|__\| | \|__\|__\| |
| COOKIES, BRIOCHES | \|__\|__\| | \|__\|__\| |
| CORNFLAKES | \|__\|__\| | \|__\|__\| |
| SNACKS | \|__\|__\| | \|__\|__\| |
| PIZZA-SNACK, WHITE PIZZA BUN (“Focaccia”) | \|__\|__\| | \|__\|__\| |
| BEANS, LENTILS, CHICKPEAS, BROAD BEANS, SOY  (boiled or canned) | \|__\|__\| | \|__\|__\| |
| FRESH COOKED PEAS | \|__\|__\| | \|__\|__\| |
| POTATOES | \|__\|__\| | \|__\|__\| |
| CARROTS | \|__\|__\| | \|__\|__\| |
| RAW TOMATOES | \|__\|__\| | \|__\|__\| |
| COOKED TOMATOES (tomato sauce; tomato puree) | \|__\|__\| | \|__\|__\| |
| GREEN SALAD | \|__\|__\| | \|__\|__\| |
| CAULIFLOWER, SPROUTS, CABBAGE, BROCCOLI | \|__\|__\| | \|__\|__\| |
| SPINACH, BEETS, CHARD, HERBS AND CATALONIA | \|__\|__\| | \|__\|__\| |
| PEPPERS | \|__\|__\| | \|__\|__\| |
| OTHER VEGETABLES  (zucchini, green beans, eggplant, artichokes, cucumbers) | \|__\|__\| | \|__\|__\| |
| CITRUS | \|__\|__\| | \|__\|__\| |
| APPLES | \|__\|__\| | \|__\|__\| |
| APRICOT, BANANAS, PEARS, PEACHES, PLUMS, GRAPES | \|__\|__\| | \|__\|__\| |
| NUTS | \|__\|__\| | \|__\|__\| |
| MELON, WATERMELON | \|__\|__\| | \|__\|__\| |
| OLIVE OIL | \|__\|__\| | \|__\|__\| |
| SEED OIL | \|__\|__\| | \|__\|__\| |
| BUTTER | \|__\|__\| | \|__\|__\| |
| MAYONNAISE | \|__\|__\| | \|__\|__\| |
| BEEF (WHITE OR RED) | \|__\|__\| | \|__\|__\| |
| CHICKEN, TURKEY, RABBIT | \|__\|__\| | \|__\|__\| |
| PORK | \|__\|__\| | \|__\|__\| |
| RAW HAM | \|__\|__\| | \|__\|__\| |
| BAKED HAM | \|__\|__\| | \|__\|__\| |
| BOLOGNA, SAUSAGE | \|__\|__\| | \|__\|__\| |
| SALAMI, AIR CURED PORK MEAT (“coppa”) | \|__\|__\| | \|__\|__\| |
| WHOLE MILK, WHOLE YOGURT | \|__\|__\| | \|__\|__\| |
| SEMI-SKIMMED MILK, LOW-FAT YOGURT | \|__\|__\| | \|__\|__\| |
| GREEN CHEESE | \|__\|__\| | \|__\|__\| |
| RIPENED CHEESE | \|__\|__\| | \|__\|__\| |
| PARMESAN CHEESE | \|__\|__\| | \|__\|__\| |
| GRATED PARMESAN CHEESE | \|__\|__\| | \|__\|__\| |
| EGGS | \|__\|__\| | \|__\|__\| |
| FRESH OR FROZEN FISH | \|__\|__\| | \|__\|__\| |
| TUNA, OTHER FISH IN OIL | \|__\|__\| | \|__\|__\| |
| CHOCOLATE | \|__\|__\| | \|__\|__\| |
| CRISP | \|__\|__\| | \|__\|__\| |
| ICECREAM | \|__\|__\| | \|__\|__\| |
| SWEET SPARKLING DRINKS | \|__\|__\| | \|__\|__\| |
| FRUIT JUICE (all types) | \|__\|__\| | \|__\|__\| |
| HONEY | \|__\|__\| | \|__\|__\| |

CONSUMPTION OF ALCOHOL/COFFEE/THE

Which of the following beverages consumed habitually and with what frequencies?

(Please tick one box for each type of beverage, indicating the number of glasses, cups per day or
per week).

| DRINK | NUMBER OF TIMES A WEEK | NUMBER OF TIMES A MONTH | OCCASIONALLY/NEVER |
| --- | --- | --- | --- |
| WINE (glasses) | \|__\| | \|__\|__\| | \|__\| |
| BEER (jugs) | \|__\| | \|__\|__\| | \|__\| |
| HARD LIQUOR | \|__\| | \|__\|__\| | \|__\| |
| COFFEE (small cups) | \|__\| | \|__\|__\| | \|__\| |
| THE (cups) | \|__\| | \|__\|__\| | \|__\| |

**Table S2.** Recommended average intake of selected food nutrients.

| **Nutrients** | **Recommended average intake** |
| --- | --- |
| Fiber, g/day | 30 |
| Protein, g/day | 58 |
| Carbohydrate, g/day | 299 |
| Lipids, g/day | 65 |
| Monounsaturated fatty acid (MUFA), g/day | 24–36 |
| Polyunsaturated fatty acid (PUFA), g/day | 7–20 |
| PUFA *n*-3, g/day | 1.25 |
| Saturated fatty acid, g/day | max 26 |
| Cholesterol , mg/day | <300 |
|  |  |
| Ascorbic acid, mg/day | 60 |
| Folic acid, µg/day | 200 |
| Alpha carotene, µg/day | - |
| Beta carotene, µg/day | 3900 |
| Carotenoids, µg/day | 6000 |
| Polyphenols and flavonoids, mg/day | - |
| Retinol, µg/day | 650 |
| Tocopherols, mg/day | 6.5 |
| Vitamin B12, µg/day | 2 |
| Vitamin D, µg/day | 5–10 |

© 2014 by the authors; licensee MDPI, Basel, Switzerland. This article is an open access article distributed under the terms and conditions of the Creative Commons Attribution license (http://creativecommons.org/licenses/by/4.0/).
